# Reconstruction of the Foot and Ankle Using Pedicled or Free Flaps: Perioperative Flap Survival Analysis

**DOI:** 10.1371/journal.pone.0167827

**Published:** 2016-12-08

**Authors:** Xiucun Li, Jianli Cui, Suraj Maharjan, Laijin Lu, Xu Gong

**Affiliations:** Department of Hand and Foot Surgery, The First Hospital of Jilin University, Changchun, Jilin Province, P.R. China; di Pompeo d'Illasi, Universita degli Studi di Roma La Sapienza Facolta di Medicina e Psicologia, ITALY

## Abstract

**Objective:**

The purpose of this study is to determine the correlation between non-technical risk factors and the perioperative flap survival rate and to evaluate the choice of skin flap for the reconstruction of foot and ankle.

**Methods:**

This was a clinical retrospective study. Nine variables were identified. The Kaplan-Meier method coupled with a log-rank test and a Cox regression model was used to predict the risk factors that influence the perioperative flap survival rate. The relationship between postoperative wound infection and risk factors was also analyzed using a logistic regression model.

**Results:**

The overall flap survival rate was 85.42%. The necrosis rates of free flaps and pedicled flaps were 5.26% and 20.69%, respectively. According to the Cox regression model, flap type (hazard ratio [HR] = 2.592; 95% confidence interval [CI] (1.606, 4.184); P < 0.001) and postoperative wound infection (HR = 0.266; 95% CI (0.134, 0.529); P < 0.001) were found to be statistically significant risk factors associated with flap necrosis. Based on the logistic regression model, preoperative wound bed inflammation (odds ratio [OR] = 11.371,95% CI (3.117, 41.478), P < 0.001) was a statistically significant risk factor for postoperative wound infection.

**Conclusion:**

Flap type and postoperative wound infection were both independent risk factors influencing the flap survival rate in the foot and ankle. However, postoperative wound infection was a risk factor for the pedicled flap but not for the free flap. Microvascular anastomosis is a major cause of free flap necrosis. To reconstruct complex or wide soft tissue defects of the foot or ankle, free flaps are safer and more reliable than pedicled flaps and should thus be the primary choice.

## Introduction

The foot and ankle are prone to injuries and diseases because of insufficient soft tissue [1]. Complex soft tissue defects of the foot and ankle caused by trauma, infection, tumor cancer or diabetes are common and can be accompanied by exposed tendons, neurovascular bundles and bone. There are multiple options for the reconstruction of complex soft tissue defects in these areas, including the use of both pedicled flaps and free flaps (e.g., a lateral supramalleolar flap [2], a peroneal artery perforator flap [3,4], posterior tibial artery perforator flap [5], a sural neurocutaneous/neurofasciocutaneous flap [6,7,8], a medial pedis flap [9], a dorsal metatarsal flap [10], a dorsalis pedis flap [11], a pedicled or free medial plantar flap [12,13], a free groin flap [14,15], a free anterolateral thigh perforator flap [16,17,18], or a free anteromedial thigh perforator flap [19]). In addition, the successful reconstruction of complex soft tissue defects of the foot and ankle is critical because of the unique standing, weight-bearing and walking functions of the foot. The management and reconstruction of soft tissue defects must thus be a surgeon’s primary focus.

Although flap transfers have become the primary method of reconstruction of complex soft tissue defects of the foot and ankle and although microsurgical techniques have advanced, skin flap failure often occurs during the perioperative period. Once skin flap failure has occurred, it can have devastating consequences. When selecting a skin flap, several factors must be considered, such as the pliability of the skin flap, the stability of standing and walking, donor site morbidity, the vascular condition at the recipient site [20], the size of the soft tissue defect, and the flap survival rate.

The purpose of this study is to determine the correlation between non-technical risk factors and the perioperative flap survival rate and to evaluate the choice of skin flap for the reconstruction of foot and ankle. In this study, the perioperative period was defined as within 2 weeks after flap transfer.

## Patients and Methods

### Data Collection Criteria and Study Design

This retrospective study was approved by the institutional review committee and ethics committee at the First Hospital of Jilin University. Written informed consent to participate in this study was obtained from each patient. The inclusion criteria were patients who had a unilateral foot or ankle injury with complex soft tissue defects and who had undergone various pedicled or free flap procedures. Only the initial pedicled or free flap procedure and its complications were studied. Patients were excluded if they had a bilateral foot or ankle injury because repeated data analysis can increase the sample error. Although secondary flap procedures were performed, the secondary transplanted flap and its complications were not the focus of our study. Considering the above-mentioned criteria, we carefully reviewed hospital records and found that 144 patients met these criteria between February 2007 and December 2014.

The characteristics of these patients, the flap transfer procedures and postoperative complications were recorded and researched. The nine risk factors assessed in this study were as follows: patient age (≤ 40 years, > 40 years), cigarette smoking (1 = smoker, 0 = nonsmoker), hypertension (1 = Yes, 0 = No), osteomyelitis (1 = Yes, 0 = No), preoperative wound bed inflammation (1 = Yes, 0 = No), trauma activation (1 = Yes, 0 = No), anatomical region (1 = hindfoot and ankle region, 2 = midfoot region, 3 = forefoot region, 4 = multiple regions), the type of flap (1 = pedicled flap, 2 = free flap), and postoperative wound infection (1 = Yes, 0 = No). The flap outcome was subdivided into flap survival and flap necrosis (1 = flap necrosis, 0 = flap survival), which were categorical variables. If free flap failure occurred within the first 48 hours, these flaps would be excluded because their failure could be more closely related to a microvascular thrombosis. A univariate analysis of the risk factors for flap necrosis was first performed. The variables that were identified as statistically significant or nearly significant were entered into a multivariate regression analysis. Diabetes mellitus could not be analyzed as a predictor because the number of patients with diabetes mellitus in this study was insufficient.

### Patient Descriptions and Management

**Fig 1:**
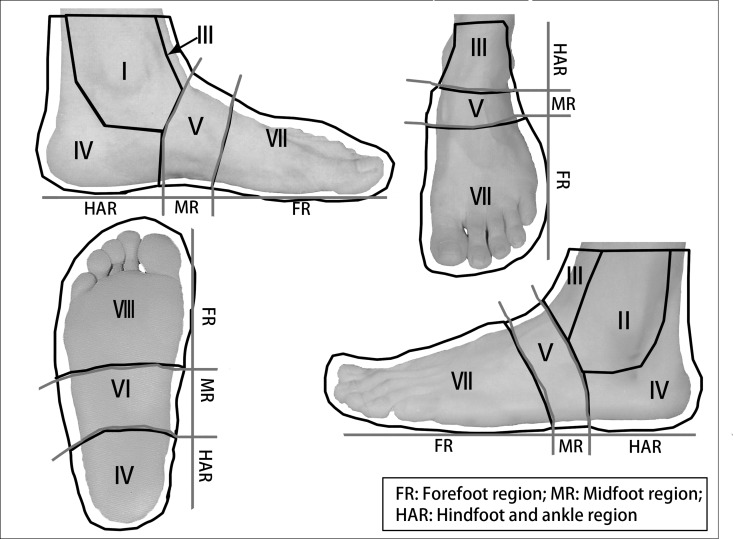
The eight subunits (I-VIII) within three regions (forefoot, midfoot, and hindfoot and ankle) of the foot and ankle.

Fourteen different flaps were used in 144 patients. Thirty patients were female, and 114 were male, with a 3.8:1 male-to-female ratio. There were 64 left-foot and 80 right-foot injuries. Comorbidities included hypertension, diabetes mellitus and osteomyelitis (**Table 1**). Trauma was the most common etiology, followed by skin ulcers and inflammation and then tumors (**Table 1**). Flap necrosis > 60% was regarded as complete necrosis (CN), and flap necrosis ≤ 60% was regarded as partial necrosis (PN) [21]. According to Godina’s study [22], the time window from trauma to 72 hours after the trauma was considered as the acute period, while the time window of the subacute stage was between 72 hours and 90 days after the trauma. In our series, 107 trauma patients underwent the flap transfer procedures in the subacute period (**Table 2**). In addition, preoperative wound bed inflammation and postoperative wound infection were identified by bacterial cultivation. Based on our clinical experience, the foot and ankle were divided into eight subunits within three regions (**Fig 1**).

**Table 1:** Patient characteristics

		Number (n = 144)	Percentage (%)
**Cigarette smoking**		44	30.56%
**Comorbidities**	Hypertension	17	11.81%
	Diabetes mellitus	7	4.86%
	Osteomyelitis	27	18.75%
**Preoperative wound bed infection**		42	29.17%
**Etiologies**	Trauma	115	79.86%
	Skin ulcers and inflammation	16	11.11%
	Post-tumor resection	8	5.56%
	Scar contracture	3	2.08%
	Diabetic foot	2	1.39%

**Table 2:** The outcome of reconstruction of the foot and ankle in trauma patients in different periods

		Total	Postoperative wound infection	Complete necrosis	Partial necrosis
**Acute period**	Free flap	2	0	0	0
	Pedicled flap	6	0	0	0
**Subacute period**	Free flap	52	8	2	1
	Pedicled flap	55	12	6	6
**Total**		115	20	8	7

K-wires, hollow screws and external fixators were used to repair fractures of the foot and ankle. Additionally, the injured tendons and major nerves were reconstructed, and negative pressure wound therapy was used. Thorough and complete debridement was carried out before the pedicled or free flap transfer. Postoperative flap care and monitoring were carried out 3–5 days after surgery, and low-molecular-weight heparin calcium was systemically administered for anticoagulation.

### Statistical Analysis

The descriptive statistics were derived from the case numbers and/or the percentages of patients. Initially, the Kaplan-Meier method coupled with a log-rank test was used for the univariate analysis to predict the relationship between the perioperative flap survival rate and the risk factors. After the univariate analysis, the variables that were identified as statistically significant or nearly significant were entered into a multivariate Cox regression model to forecast the main risk factor for perioperative flap necrosis. The relationship between postoperative wound infection and the risk factors was also analyzed using a binary logistic regression model. P values < 0.05 indicated statistical significance. All statistical analyses were performed using IBM SPSS 21.0 software (IBM Corp., Armonk, N.Y., U.S.A.).

## Results

### Perioperative Complications

**Fig 2:**
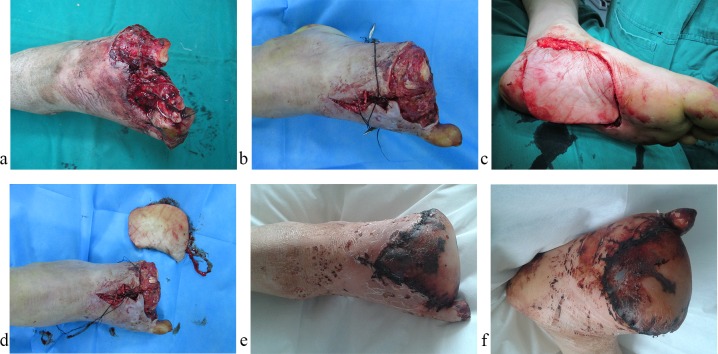
A 49-year-old male patient suffered from a mutilating forefoot injury, and the forefoot was reconstructed using the contralateral free plantar medial flap. a, Mutilating forefoot injury; b, after forefoot debridement; c and d, design and harvesting of free medial plantar flap; e and f, PN of the flap on the sixth postoperative day.

The average age at the time of flap surgery was 37.9 years (range, 3–74 years). In the trauma patient group, the mean interval between injury and flap transfer was 13.7 days (range, 8 hours to 63 days), and the rates of postoperative wound infection and flap necrosis in the subacute stage were 18.69% and 14.02%, respectively (**Table 2**). The flap dimensions ranged from 4×3 cm to 35×20 cm. Postoperative wound infections were observed in 27 patients. The donor sites for 94 patients underwent full-thickness skin grafts, which survived completely. Wound dehiscence at the donor site was observed in three patients. Five of 57 free flaps required re-exploration within the first 24–48 hours because of arterial thrombosis at the microvascular anastomosis site. Three free flaps were successfully salvaged, while two free flaps with arterial thrombosis experienced complete necrosis within the first 24–48 hours. In addition, one free flap exhibited PN on the sixth postoperative day (**Fig 2**E and **2**F). Eleven pedicled flaps experienced PN, and seven pedicled flaps exhibited CN. The overall rate of pedicled and free flap survival was 85.42% (123 of 144 cases). The necrosis rates of the free flaps and pedicled flaps were 5.26% (3 of 57 cases) and 20.69% (18 of 87 cases), respectively (**Table 3**). Of the 21 patients with flap necrosis, a secondary flap transfer was performed in four cases, while 10 patients underwent a full-thickness skin graft, and the remaining patients healed with local wound care. In all other patients, all wounds healed without complications. **Table 3** shows the results for the 144 skin flaps.

**Table 3:** The results of the 144 skin flaps

Flap type		Number	Flap size (cm^2^)	Preoperative wound bed inflammation	Complete necrosis	Partial necrosis	Postoperative wound infection	FTSG
**Pedicled flap**	Antemalleolar flap	1	5×4	0	0	0	0	1
	Dorsalis pedis flap	3	6×4 to 7×6	1	0	1	1	3
	First dorsal metatarsal artery flap	3	4×3 to 10×3	0	0	0	1	3
	Gastrocnemius myocutaneous flap	1	10×5	1	1	0	1	1
	Lateral supramalleolar flap	4	7×4 to 8×5	2	0	0	1	2
	Medial pedis flap	1	8×7	0	0	0	0	1
	Medial plantar flap	6	4×3 to 7×6	2	0	1	1	6
	Medial supramalleolar flap	6	5.5×4 to 12×5	1	0	1	2	4
	Peroneal artery perforator flap	15	7×7 to 25×8	4	3	3	4	12
	Posterior tibial artery perforator flap	9	5×3 to 17×8	3	1	2	1	6
	Sural neurocutaneous flap	38	5×3.5 to 25×12	11	2	3	6	21
	**Total pedicled flap (%)**	**87**		**25(28.74%)**	**7(8.05%)**	**11(12.64%)**	**18(20.69%)**	**60**
**Free flap**	Free ALT perforator flap	49	7×4 to 35×20	15	2	0	9	30
	Free AMT perforator flap	1	16×11	0	0	0	0	1
	Free groin flap	4	10×7 to 12×8	1	0	0	0	0
	Free medial plantar flap	3	8×5 to 13×7	1	0	1	0	3
	**Total free flap (%)**	**57**		**17(29.82%)**	**2(3.51%)**	**1(1.75%)**	**9(15.79%)**	**34**
**Total (%)**		**144**		**42(29.17%)**	**9(6.25%)**	**12(8.33%)**	**27(18.75%)**	**94**

FTSG: Full-thickness skin graft. ALT: Anterolateral thigh. AMT: Anteromedial thigh.

### Analysis of Risk Factors Associated with Flap Necrosis

Two free flap failures were excluded because their failure occurred within the first 48 postoperative hours. One hundred forty-two flaps were used to analyze the non-technical risk factors associated with flap necrosis.

**Fig 3:**
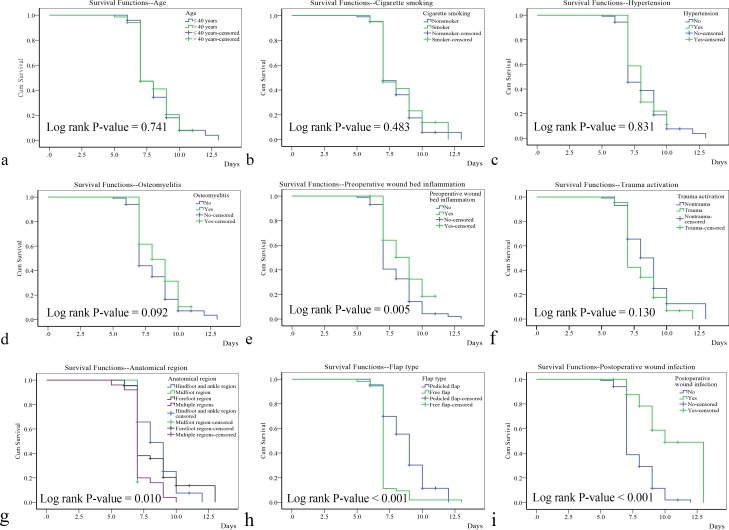
Overall survival. The outcomes of the univariate analysis of risk factors influencing the perioperative flap survival rate. The univariate analysis was performed using the Kaplan-Meier method coupled with a log-rank test.

**Fig 3** shows the relationship between the perioperative flap survival rate and the risk factors. In the univariate analysis, preoperative wound bed inflammation (P = 0.005), anatomical region (P = 0.010), flap type (P < 0.001) and postoperative wound infection (P < 0.001) were statistically significant risk factors. A multivariate Cox regression model was used to analyze the statistically significant variables found by the univariate analysis. Based on the results of the multivariate Cox model (**Table 4**), flap type (hazard ratio [HR] = 2.592; 95% confidence interval [CI] (1.606, 4.184); P < 0.001) and postoperative wound infection (HR = 0.266; 95% CI (0.134, 0.529); P < 0.001) were found to be statistically significant factors. However, as shown in **Table 5**, in the free flap group, postoperative wound infection (HR = 0.509; 95% CI (0.184, 1.405); P = 0.192) was not a statistically significant risk factor for free flap necrosis, whereas in the pedicled flap group (**Table 6**), postoperative wound infection (HR = 0.202; 95% CI (0.071, 0.578); P = 0.003) was a statistically significant risk factor.

**Table 4:** Multivariate Cox model results for predictive factors influencing flap survival rate (n = 142)

	Coefficient	P—value	HR	HR 95.0% CI	
				Lower	Upper
**Osteomyelitis**	0.203	0.618	1.225	0.552	2.719
**Preoperative wound bed inflammation**	-0.204	0.569	0.815	0.404	1.646
**Anatomical region**	-0.017	0.851	0.983	0.825	1.173
**Flap type**	0.953	***< 0*.*001***	2.592	1.606	4.184
**Postoperative wound infection**	-1.325	***< 0*.*001***	0.266	0.134	0.529

HR: hazard ratio. CI: confidence interval.

**Table 5:** Multivariate Cox model results for predictive factors of free flap (n = 55) necrosis

	Coefficient	P-value	HR	HR 95.0% CI	
				Lower	Upper
**Preoperative wound bed inflammation**	0.078	0.819	1.082	0.551	2.122
**Anatomical region**	0.006	0.967	1.006	0.749	1.352
**Postoperative wound infection**	-0.676	0.192	0.509	0.184	1.405

HR: hazard ratio. CI: confidence interval.

**Table 6:** Multivariate Cox model results for predictive factors of pedicled flap (n = 87) necrosis

	Coefficient	P-value	HR	HR 95.0% CI	
				Lower	Upper
**Preoperative wound bed inflammation**	-0.276	0.378	0.759	0.410	1.402
**Anatomical region**	-0.017	0.884	0.983	0.786	1.230
**Postoperative wound infection**	-1.597	***0*.*003***	0.202	0.071	0.578

### Risk Factors Associated with Postoperative Wound Infection

As shown in **Table 7**, preoperative wound bed inflammation (odds ratio [OR] = 11.371; 95% CI (3.117, 41.478); P < 0.001) was found to be a statistically significant risk factor for postoperative wound infection. No other analyzed risk factors were found to be statistically significant.

**Table 7:** Binary logistic regression model for risk factors associated with postoperative wound infection (n = 144)

	Coefficient	P value	OR	OR 95.0% CI	
				Lower	Upper
**Age**	-0.002	0.923	0.998	0.966	1.032
**Cigarette smoking**	0.662	0.200	1.938	0.704	5.333
**Hypertension**	0.076	0.917	1.079	0.255	4.560
**Osteomyelitis**	-0.249	0.715	0.779	0.204	2.976
**Trauma activation**	0.062	0.923	1.064	0.300	3.783
**Anatomical region**	-0.087	0.745	0.917	0.543	1.547
**Preoperative wound bed inflammation**	2.431	***< 0*.*001***	11.371	3.117	41.478
**Flap types**	-0.373	0.577	0.689	0.186	2.547

OR: odds ratio. CI: confidence interval.

## Discussion

It was assumed that predictive variables would affect perioperative flap survival. **Fig 3** indicates that preoperative wound bed inflammation, anatomical region, flap type and postoperative wound infection specifically affect the perioperative flap survival rate. In the multivariate Cox regression model (**Table 4**), flap type and postoperative wound infection were both independent risk factors influencing flap survival. Flap type and postoperative wound infection were associated with HRs of 2.592 and 0.266, respectively. Considering their HRs, the major risk factor influencing the perioperative flap survival rate was flap type.

**Table 5** shows that postoperative wound infection is not a risk factor of free flap necrosis, whereas, Wong et al. [23] reported that operative time is a significant risk factor for free flap loss. However, in our series, five free flaps occurred arterial thrombosis within the first 24–48 hours. Among them, two free flaps exhibited CN within the first 24–48 hours. These flap failures could be more closely related to arterial thrombosis than to infection because infection can result in arterial thrombosis at a later stage (e.g., 3 days later) after complete and thorough debridement. Therefore, we agree that microvascular anastomosis is a major cause of free flap necrosis.

In contrast, as shown in **Table 6**, postoperative wound infection is a risk factor associated with pedicled flap necrosis. Infection gives rise to an inflammatory reaction, and the release of inflammatory mediators can lead to the formation of thrombi or vasospasms and can thus damage blood vessels [24,25]. Surgical site infection can also occur due to the complex effects of both bacterial colonization and host defense mechanisms, resulting in an increased risk of vascular surgery failure [24]. Postoperative wound infection is thus a risk factor of pedicled flap necrosis. However, Bekara et al. agree that the significant risk factors for pedicled flap complications in the lower limb include age older than 60 years, diabetes, and arteriopathy [26]. Wei et al. believe that flap-related factors may be closely related to pedicled flap necrosis; namely, the length of the pedicled flap and the width of the pedicled flap around the perforator pedicle may lead to a venous drainage problem and an insufficient arterial blood supply in a large pedicled flap by affecting the number of choke vessels between angiosomes or inclusive linking vessels between perforasomes [27]. Thus, pedicled flap necrosis is not only related to postoperative wound infection but also associated with flap-related factors.

Regarding risk factors associated with postoperative wound infection, according to **Table 7**, preoperative wound bed inflammation was a risk factor for postoperative wound infection. According to Godina’s study [22], the treatment of complex lower limb traumas in the acute period (< 72 hours) or in the chronic period after multiple debridements (> 90 days) reduces the risk of recipient site infection and osteomyelitis. In addition, that study reported that the postoperative infection rates for flap transfer in the acute, subacute, and chronic stages were 1.5%, 17.5% and 6%, respectively. However, in the trauma patient group in the current study, the postoperative wound infection rate in the subacute stage was 18.69% (20 of 107 cases) (**Table 2**), strongly suggesting the incidence of preoperative recipient site inflammation (29.17%), which was possibly responsible for postoperative wound infection (18.75%). Therefore, to increase the perioperative flap survival rate, multiple thorough debridements should be performed before flap transfer.

With regard to flap selection for reconstruction of the foot and ankle, Bekara et al. agree that the success of free flaps and pedicled flaps in reality appears similar [28]. Gir et al. believe that pedicled perforator flap use is a safe and reliable procedure for reconstructing soft tissue defects [29]. Xiong et al. [30] and Kang et al. [31] think that free flap use in foot and ankle reconstruction is safe and reliable due to very robust vascularization and a low risk of infection. However, in our study, **Fig 3** and **Table 4** indicate that flap type significantly affects the perioperative flap survival rate in the foot and ankle. Zhu et al. report that the overall rate of pedicled and free flap survival is 77.9% and that the necrosis rates of pedicled and free flaps are 17.7% and 7.9%, respectively [32]. Additionally, Bekara et al. indicate that the overall pedicled flap survival rate is 84.3% [26], and Hollenbeck et al. found an overall primary free flap survival rate of 92% [1]. In our series, the complete survival rate of free flaps (94.74%, 54 of 57 cases) was greater than that of pedicled flaps (79.31%, 69 of 87 cases) (**Table 3**). Therefore, for the reconstruction of complex or wide soft tissue defects of the foot or ankle, we agree that the use of free flaps is safer and more reliable than the use of pedicled flaps.

For soft tissue defects in the hindfoot and ankle regions, pedicled peroneal artery perforator flaps, posterior tibial artery perforator flaps and sural neurocutaneous flaps are frequently used because these flaps’ dimensions are large enough to reconstruct a single subunit. When reconstructing these subunits, either free flaps or pedicled flaps can be used. However, a study by Pinsolle et al. indicates that pedicled flaps should be the first choice when reconstructing soft tissue defects in these areas but that free flaps should be used for oversized or composite defects [33]. In the midfoot region (**Fig 2**), soft tissue defects of subunit VI are not common, and it is difficult to cover soft tissue defects in this subunit with a pedicled flap from the leg and dorsal pedis because of the transfer distance. Moreover, in this region, when bone is not involved, reconstruction is accomplished with a full-thickness skin graft, rather than a complex flap, due to the low functional demand. In subunit V, an isolated soft tissue defect can be reconstructed using either a free flap or a pedicled flap because of the small size of this subunit. With regard to reconstruction of the forefoot, the reconstruction must consider the unique characteristics of this region. In particular, the forefoot is situated at the most distal region of the foot, so the transfer distance for a pedicled flap from the leg and foot is too great to fully cover large defects in the forefoot region. Because this area is usually covered by a shoe, reconstruction also requires a thin flap. Therefore, we agree that a free anterolateral thigh flap is a good choice for the reconstruction of complex or wide soft tissue defects of the forefoot.

There are some limitations to this research. First, the patient population exhibited a certain heterogeneity. Second, non-technical variables related to flap loss were researched, and flap loss is associated with both flap factors and microvascular problem. Third, identifying preoperative wound bed inflammation and postoperative wound infection via bacterial cultivation is likely to produce some false positives or false negatives result. Finally, the outcome of this research requires further validation in a larger set of patients, preferably in a multi-center study.

## Conclusions

Flap type and postoperative wound infection were both independent risk factors influencing the flap survival rate in the foot and ankle. However, postoperative wound infection was a risk factor for the pedicled flap but not for the free flap. In addition, pedicled flap necrosis may be associated with flap-related factors. Microvascular anastomosis is a major cause of free flap necrosis. Preoperative wound bed inflammation was found to be a risk factor associated with postoperative wound infection. To increase the perioperative flap survival rate, multiple thorough debridements should be performed before flap transfer. For the reconstruction of complex or wide soft tissue defects of the foot or ankle, free flaps are safer and more reliable than pedicled flaps and should thus be the primary choice.

## Supporting Information

S1 Fig The eight subunits (I-VIII) within three regions (forefoot, midfoot, and hindfoot and ankle) of the foot and ankle. (XLSX)

S2 Fig A 49-year-old male patient suffered from a mutilating forefoot injury, and the forefoot was reconstructed using the free contralateral plantar medial flap. a, Mutilating forefoot injury; b, after forefoot debridement; c and d, design and harvesting of free medial plantar flap; e and f, PN of the flap on the sixth postoperative day.
(XLSX)

S3 Fig Overall survival. The outcomes of the univariate analysis of risk factors influencing the perioperative flap survival rate. The univariate analysis was performed using the Kaplan-Meier method coupled with a log-rank test.
(XLSX)

S1 Table Patient characteristics (XLSX)

S2 Table The outcome of reconstruction of the foot and ankle in trauma patients in different periods (XLSX)

S3 Table The results for the 144 skin flaps (XLSX)

S4 Table Multivariate Cox model results for predictive factors influencing flap survival rate (n = 142) (XLSX)

S5 Table Multivariate Cox model results for predictive factors of free flap (n = 55) necrosis (XLSX)

S6 Table Multivariate Cox model results for predictive factors of pedicled flap (n = 87) necrosis (XLSX)

S7 Table Binary logistic regression model for risk factors associated with postoperative wound infection (n = 144) (XLSX)
